# Evidence For Cannabidiol Modulation of Serotonergic Transmission in a Model of Osteoarthritis via *in vivo* PET Imaging and Behavioral Assessment

**DOI:** 10.23958/ijirms/vol07-i06/1418

**Published:** 2022-06-03

**Authors:** Yu-Shin Ding, Jiacheng Wang, Vinay Kumar, James Ciaccio, Sami Dakhel, Cathy Tan, Jonathan Kim, Sabrina Lee, Hilla Katz-Lichtenstein, Zakia Gironda, Orin Mishkit, Jakub Mroz, Raul Jackson, Grace Yoon, Begona Gamallo-Lana, Molly Klores, Adam Mar

**Affiliations:** 1 Radiology, New York University School of Medicine, New York, NY, USA,; 2 Psychiatry, New York University School of Medicine, New York, NY, USA,; 3 Chemistry, Fordham University, Bronx, NY, USA,; 4 Chemistry, New York University, New York, NY, USA,; 5 Rodent Behavioral Core, New York University School of Medicine, New York, NY, USA.

**Keywords:** Cannabidiol, Pain, PET imaging, Anxiety, Serotonin receptor, Osteoarthritis

## Abstract

**Background:**

Preclinical studies indicate that cannabidiol (CBD), the primary nonaddictive component of cannabis, has a wide range of reported pharmacological effects such as analgesic and anxiolytic actions; however, the exact mechanisms of action for these effects have not been examined in chronic osteoarthritis (OA). Similar to other chronic pain syndromes, OA pain can have a significant affective component characterized by mood changes. Serotonin (5-HT) is a neurotransmitter implicated in pain, depression, and anxiety. Pain is often in comorbidity with mood and anxiety disorders in patients with OA. Since primary actions of CBD are analgesic and anxiolytic, in this first *in vivo* positron emission tomography (PET) imaging study, we investigate the interaction of CBD with serotonin 5-HT_1A_ receptor via a combination of *in vivo* neuroimaging and behavioral studies in a well-validated OA animal model.

**Methods:**

The first aim of this study was to evaluate the target involvement, including the evaluation of modulation by acute administration of CBD, or a specific target antagonist/agonist intervention, in control animals. The brain 5-HT_1A_ activity/availability was assessed via *in vivo* dynamic PET imaging (up to 60 min) using a selective 5-HT_1A_ radioligand ([^18^F]MeFWAY). Tracer bindings of 17 ROIs were evaluated based on averaged SUVR values over the last 10 min using CB as the reference region. We subsequently examined the neurochemical and behavioral alterations in OA animals (induction with monosodium iodoacetate (MIA) injection), as compared to control animals, via neuroimaging and behavioral assessment. Further, we examined the effects of repeated low-dose CBD treatment on mechanical allodynia (von Frey tests) and anxiety-like (light/dark box tests, L/D), depressive-like (forced swim tests, FST) behaviors in OA animals, as compared to after vehicle treatment.

**Results:**

The tracer binding was significantly reduced in control animals after an acute dose of CBD administered intravenously (1.0 mg/kg, i.v.), as compared to that for baseline. This binding specificity to 5-HT_1A_ was further confirmed by a similar reduction of tracer binding when a specific 5-HT_1A_ antagonist WAY1006235 was used (0.3 mg/kg, i.v.). Mice subjected to the MIA-induced OA for 13–20 days showed a decreased 5-HT_1A_ tracer binding (25% to 41%), consistent with the notion that 5-HT_1A_ plays a role in the modulation of pain in OA. Repeated treatment with CBD administered subcutaneously (5 mg/kg/day, s.c., for 16 days after OA induction) increased 5-HT_1A_ tracer binding, while no significant improvement was observed after vehicle. A trend of increased anxiety or depressive-like behavior in the light/dark box or forced swim tests after OA induction, and a decrease in those behaviors after repeated low-dose CBD treatment, are consistent with the anxiolytic action of CBD through 5HT_1A_ receptor activation. There appeared to be a sex difference: females seem to be less responsive at the baseline towards pain stimuli, while being more sensitive to CBD treatment.

**Conclusion:**

This first *in vivo* PET imaging study in an OA animal model has provided evidence for the interaction of CBD with the serotonin 5-HT_1A_ receptor. Behavioral studies with more pharmacological interventions to support the target involvement are needed to further confirm these critical findings.

## 1. Background

Chronic pain is a major public health issue, affecting >20% of adults (Dahlhamer et al., 2018a; Dahlhamer et al., 2018b) and costing >$600 billion annually. (Gaskin and Richard, 2012; Nahin, 2015) Nearly 27 million US adults have osteoarthritis (OA), a chronic and progressive disease for which pain is the primary symptom, making it a common cause of chronic pain. (Lawrence et al., 2008)

Current pharmacologic therapies for OA are limited and have significant side-effect profiles. Acetaminophen (Towheed et al., 2006) or non-steroidal anti-inflammatory drugs (NSAIDs) are modestly effective but carry hepatotoxicity, cardiovascular, gastrointestinal and renal risks. (Bannuru et al., 2015) For refractory pain, recommendations include opioids, which carry significant risks for dependence and overdose death from respiratory depression. (Qaseem et al., 2017) The opioid epidemic resulting from a false claim of the low addictive potential of opioid analgesics has created an urgent need for novel, effective, and non-addictive treatments for highly prevalent, disabling, and refractory pain disorders. (Towheed et al., 2006; Lawrence et al., 2008; Gaskin and Richard, 2012; Bannuru et al., 2015; Nahin, 2015; DeMik et al., 2017; Dahlhamer et al., 2018a; Seth et al., 2018)

Although both THC and CBD exhibit antinociceptive effects,(Devinsky et al.),(Wade et al., 2003; Barichello et al., 2012; Fallon et al., 2017; Meng et al., 2017; Cunetti et al., 2018; Stockings et al., 2018) CBD holds particular promise as it lacks psychoactive and addictive properties.(Babalonis et al., 2017) CBD has been shown to exhibit a wide range of pharmacological effects (e.g., anticonvulsant, analgesic, anxiolytic, anti-inflammatory, hypnotic, antipsychotic and neuroprotective actions);(Guimaraes et al., 1990; Zuardi et al., 1991; Moreira and Guimaraes, 2005; Long et al., 2006; Moreira et al., 2006; Resstel et al., 2006; Lemos et al., 2010; Zanelati et al., 2010; Campos et al., 2012; Uribe-Marino et al., 2012; Hassan et al., 2014) however, the exact mechanisms of action for these effects have not been examined in OA.

OA is a disease of the entire joint, causing cartilage degeneration, bone remodeling, and inflammation.(Loeser et al., 2012),(Hawker et al., 2008) OA pain is considered nociceptive, but some OA patients have neuropathic pain.(Hochman et al., 2013; Thakur et al., 2014) Pain mechanisms involve peripheral and central sensitization and inflammation.(Schuelert and McDougall, 2009; Parks et al., 2011; Havelin et al., 2016; Miller et al., 2017) Neuroimaging can be used to objectively assess the mechanisms of action related to the efficacy of analgesics as a quantitative and complementary measure to subjective pain reporting. The use of positron emission tomography (PET), which has a greater specificity than other imaging modalities, is particularly critical for determining target engagement and identifying the mechanisms underlying potential contributions of CBD to pain relief and functional restoration in chronic pain. Several targets, including CB1, 5-HT_1A_, FAAH, TSPO and TRPV1, have been suggested to be activated or modified after CBD administration.(Bisogno et al., 2001; Costa et al., 2004; Russo et al., 2005; Costa et al., 2007; Ryberg et al., 2007; Thomas et al., 2007; De Petrocellis et al., 2008; Maione et al., 2011; Ortega-Alvaro et al., 2011; Campos et al., 2012; Xiong et al., 2012; Ward et al., 2014; Ortega-Alvaro et al., 2015; Philpott et al., 2017; Rodriguez-Munoz et al., 2018) Similar to other chronic pain syndromes, OA pain can have a significant affective component characterized by mood changes. Serotonin (5-HT) is a neurotransmitter implicated in pain,(Wolfe et al., 1997; Bardin et al., 2000) depression, and anxiety.(Lesch et al., 1996; Ressler and Nemeroff, 2000; Holmes et al., 2003) Pain is often in comorbidity with mood and anxiety disorders in OA patients.(Rosemann et al., 2007; Axford et al., 2010) Since analgesic and anxiolytic are primary actions of CBD, in this first in vivo PET imaging study using the most promising and selective 5-HT_1A_ receptor ligand, [^18^F]trans-MeFWAY ([^18^F]MeFWAY), (Saigal et al., 2006; Wooten et al., 2011; Choi et al., 2013; Saigal et al., 2013) we investigate the interaction of CBD with 5-HT_1A_ receptor via a combination of in vivo neuroimaging and behavioral studies in a well-validated OA animal model. These sets of neuroimaging and behavioral studies designed to identify the most valid mechanistic marker attributed to the therapeutic effects of CBD will guide us to fine-tune further mechanistic studies for future clinical trials in patients with OA. Thus, the first aim of this study was to evaluate the target involvement, including the evaluation of modulation by acute administration of CBD, alone or in combination with specific antagonist/agonist for target drug intervention in control animals. We subsequently examined the neurochemical and behavioral alterations in OA animals, as compared to control animals, via neuroimaging studies and behavioral assessment. Further, we examined the effects of repeated low-dose CBD treatment on mechanical allodynia and anxiety and depressive-like behaviors in OA mice, as compared to those after placebo treatment.

## 2. Methods

### 2.1. Animals

Adult mice (C57BL/6J, Charles River, New York) were used (males and females, 8–10 weeks old and weighing 20 to 26 g on arrival) and housed in groups of 4 or 5 in standard polycarbonate cages under standard laboratory conditions (12-hour light–dark cycle; temperature at 20 ± 2°C; 50%–60% relative humidity). All in vivo PET/CT imaging studies were conducted during the light phase between 12:00 and 18:00. All behavioral experiments were conducted during the light phase between 14:00 and 18:00. Repeated drug injections were given between 12:00 to 14:00. Animals were placed in the imaging or behavioral rooms at least 1 hour prior to studies for habituation. All animal procedures were approved by the New York University Medical School Institutional Animal Care and Use Committees and performed in accordance with the National Institutes of Health Animal Care Guidelines.

### 2.2. OA Animal model: induction with MIA injection

Osteoarthritis (OA) was chemically induced by a single injection of monosodium iodoacetate (MIA, 0.5–1.0 mg in saline) into the right knee joint, a well-validated OA model with a rapid onset (1–2 week). (Micheli et al., 2019),(McDougall et al., 2017),(Longo et al., 2012; Vincent et al., 2012; Pitcher et al., 2016) Solutions of MIA in sterile saline (0.9% NaCl) at the desired concentrations were freshly prepared on the day of the injection. Based on our pilot studies in mice, 1 mg of MIA in 10 μL saline produced good results, consistent with previously recommended dose. (Pitcher et al., 2016) Briefly, under isoflurane anesthesia (3% induction; 1.5% maintenance), the area surrounding the knee joint was trimmed and wiped with alcohol until the patellar tendon (white line below the patella) became visible. A Hamilton syringe with a 26 G needle was used for MIA injections. Control animals did not undergo this surgery. After recovery from injections, mice were housed separately in groups of 4 or 5.

### 2.3. Radiotracer ([^18^F]MeFWAY) preparation

#### 2.3.1. Precursor preparation: Preparation of trans-[^18^F]MeFWAY tosyl precursor and reference compound trans-MeFWAY

**Figure 1:**
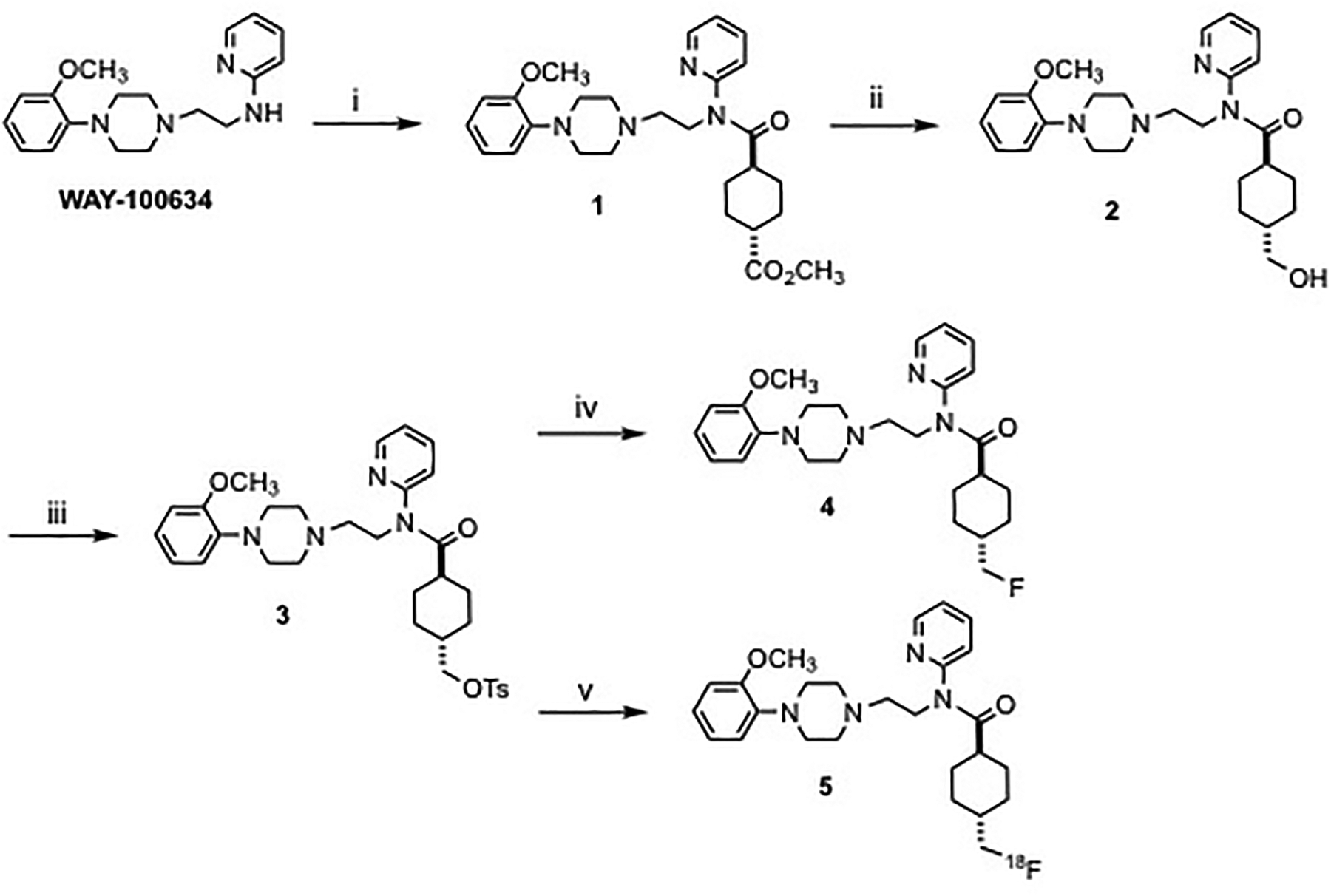
Synthesis Scheme of [^18^F]MeFWAY: Synthesis scheme of [^18^F]MeFWAY: (i) methyl *trans*-4-(chlorocarbonyl)cyclohexane-1-carboxylate, CH_2_Cl_2_, rt, 69%; (ii) NaBH_4_, EtOH, rt, 2 days, 40–60%; (iii) TsOTs, Et_3_N, CH_2_Cl_2_, rt, 50–55%; (iv) DAST, CH_2_Cl_2_, rt, 20–40%; (V) [^18^F]fluoride, Kryptofix222, K_2_CO_3_

As shown in Figure 1, the tosyl precursor trans-N-[2-[4-(2-methoxyphenyl) −1-piperazinyl] ethyl]- 4-[[[(4-methylphenyl) sulfonyl] oxy] methyl]-N-2-pyridinylcyclohexanecarboxamide (3) and the corresponding reference compound MeFWAY (4) were prepared from WAY-100634 by methods previously described, (Choi et al., 2010; Achanath et al., 2015) with the exception of the intermediate 2, which was prepared by chemoselective reduction of 1 using sodium borohydride in ethanol at room temperature while monitoring reaction progress by TLC (for a similar reduction, see reference (Din Belle et al., 2018)). Products were purified by either flash column chromatography or dry column vacuum chromatography on silica gel. (Pedersen and Rosenbohm, 2001) Spectroscopic data were consistent with previous reports, as determined by 1H NMR (400 MHz) and 13C NMR (100 MHz) analyses.

#### 2.3.2. Radiotracer preparation

All chemicals and solvents, such as Kryptofix 2.2.2^®^ for synthesis (≥ 99%); potassium carbonate (anhydrous, ≥ 99%); acetonitrile (MeCN, anhydrous, 99.8%); methanol (HPLC grade, ≥ 99.9%), were purchased from Sigma-Aldrich (St. Louis, MO, USA). Sterile water for Injection (USP) was purchased from Hospsira, INC (Lake Forest, IL, USA). Sterile ethanol (200 proof anhydrous, USP) was purchased from Decon Laboratories, Inc (King of Prussia, PA, USA). Sodium chloride (0.9%, USP) was purchase from Fresenius Kabi (Lake Zurich, IL, USA). Millex^®^ GV filter 0.22 μm PVDF was purchased from Merck Millipore Ltd (Burlington, MA, USA). Sep-Pak^®^ C18 plus Cartridge and Sep-Pak^®^ Light, Waters Accell^™^ Plus QMA Cartridge were purchased from Waters (Milford, MA, USA).

Eclipse-HP cyclotron 11 MeV proton beam (Siemens, Munich, Germany) was used for [^18^F]fluoride production. The F-18 radiolabeling process was carried out on a GE Tracerlab FXFN auto module (GE Medical Systems, Germany). For [^18^F]MeFWAY purification, HPLC separation was carried out with the TRACERlab FXFN synthesis module built-in HPLC system equipped with a UV detector and a radioactivity detector and semi-preparative RP18 Phenomenex Luna 10μm 250 X 10mm column that was purchased from Phenomenex, Inc (Torrance, CA, USA). For quality control, analysis was carried out on Phenomenex RP18 Luna 5μm 250 X 4.60mm; 5-micron column (Phenomenex, Inc, Torrance, CA, USA). Quality control HPLC system (Prominence UV/Vis detector, SPD-20A; Communication Bus module, CBM-20A; Prominence Liquid Chromatography LC) was purchased from Shimadzu Scientific Instruments, Inc (Riverwood Drive Columbia, MD, USA). Flow-Count radio HPLC detector system was purchased from Eckert & Ziegler Radiopharm. Inc (Hopkinton, MA, USA). The measurement of radioactivity was determined with CRC 55tR PET dose calibrator (Capintec, Ramsey, NJ, USA).

Enriched [^18^O] H_2_O water in a 2.4 mL target was irradiated with protons (proton energy 11 MeV) exploiting the ^18^O(p,n)^18^F nuclear reaction to produce [^18^F]F^−^ with a beam current of 60 μA and a total bombardment time of 30 min. After irradiation, the [^18^O]H_2_O containing [^18^F]F^−^ was transferred in a pneumatic transport system from the cyclotron to the radiopharmaceutical laboratory. The [^18^F]F^−^ was separated from the [^18^O]H_2_O using an anion exchange cartridge (Sep-Pak^®^ Light, Waters Accell^™^ Plus QMA Cartridge) in the HCO_3_^−^ form.

#### 2.3.3. Automated Radiosynthesis of [^18^F]trans-MeFWAY ([^18^F]MeFWAY)

[^18^F]MeFWAY (**5**) was synthesized using a modified procedure described previously.(Saigal et al., 2006; Wooten et al., 2011; Choi et al., 2013; Saigal et al., 2013) We introduced the fully automated synthesis that was carried out on the GE TRACERlab FXFN synthesizer via a simplified one-step one-pot procedure as shown in Figure 1. Before delivery of [^18^F]fluoride, the synthesizer was set up as follows: **Vial 1** was loaded with a mixture of Kryptofix 2.2.2 (4 mg), K_2_CO_3_ (1 mg), methanol (0.9 mL) and water (0.1 mL); **Vial 2** with acetonitrile (1 mL); **Vial 3** with *trans*-tosylated precursor (1 mg) dissolved in anhydrous MeCN (0.5 mL); **Vial 4** with sterile water (1.5 mL); **Vial 7** with saline (sodium chloride 0.9%, 9 mL); **Vial 8** with sterile ethanol (1 mL); **Vial 9** with sterile water (10 mL); and the round bottom flask was loaded with sterile water (15 mL).

After the delivery of [^18^F] fluoride from the cyclotron to the synthesizer, the radioactivity passed through an anion exchange Sep-Pak^®^ Light, Waters Accell^™^ Plus QMA Cartridge (preconditioned with K_2_CO_3_ (1 M, 5 mL), followed by water (5 mL)), where [^18^F]fluoride was trapped and [^18^O]water was collected for recycling. The trapped [^18^F]fluoride was eluted off from the Sep-PaK QMA cartridge into the reactor vessel with a solution of K222/K_2_CO_3_ (1.0 mL; Vial 1). Acetonitrile (Vial 2) was added into the reactor vessel and the solvent was evaporated at 110°C for 5 min under a stream of helium gas, followed by vacuum drying for 5 min to form [K_222_]^+18^F^−^ complex. After cooling down the reactor to 70°C, the solution of *trans*-tosylated precursor **3** from vial 3 was added to the aforementioned dried [K_222_]^+18^F^−^ complex. The reaction mixture was heated at 100°C for 5 min. The reactor was cooled to 60°C before water (1.5 mL, Vial 4) was added. The crude product was transferred to the HPLC tube for loop injection and purified on a semi-preparative reverse phase column (Phenomenex Luna 10μm 250 X 10mm; RP18 column; mobile phase 45% CH_3_CN/H_2_O containing 0.1% trimethylamine; flow rate 5 mL/min; wavelength = 254 nm). The labeled product with an approximate retention time of 17.8 minutes was collected into the round bottom flask containing 15 mL sterile water. The resulting solution was passed through a Sep-Pak^®^ C18 Plus cartridge that had been preconditioned with ethanol (5 mL) followed by water (10 mL). The C18 cartridge containing labeled product was washed with water (10 mL; Vail 9), and then eluted to the product vial with ethanol (1 mL; Vial 8) followed by elution with saline for injection (9 mL; Vial 7). Final tracer solution was passed through a 0.22 μm sterile MillexW GV filter to a sterile dose vial (30 mL size) for use in the PET studies after passing quality control evaluation.

### 2.4. MicroPET/CT imaging and data analysis

All animals were fasted for at least 4 hours before scans. Before imaging, mice were anesthetized with isoflurane (2–3%) and cannulated in tail vein using home-made cannulas that were prepared using 30 G needles (Exel International Hypodermic) and 2” tubing (Intramedic^™^ PE Tubing, 0.011” ID). Mice were then taped in a flat, prone position with arms at sides in a home-made mouse holder. Each isoflurane-anesthetized mouse was injected with [^18^F]MeFWAY (0.3–0.7 mCi in 100–200 μL saline) via tail vein and scanned for 30–60 min using a high-resolution microPET/CT imaging (Inveon, Siemens). The list mode PET data were collected dynamically, and rebinned using a Fourier rebinning algorithm. The CT scan performed right after the PET scan facilitated the attenuation correction and anatomical co-registration.

#### Data analysis:

To investigate the interaction of CBD with the serotonin 5-HT_1A_ receptor, tracer bindings of [^18^F]MeFWAY in control animals were compared at the baseline (injection with tracer only), or after pretreatment of an acute dose of either CBD (1.0 mg/kg, i.v.) or the specific 5-HT_1A_ antagonist WAY1006235 (0.3 mg/kg, i.v.), at 5 min prior to tracer injection. Tracer bindings after OA induction (at day 13 and day 20 post MIA injection) were also compared to their baseline tracer bindings. Group comparison on tracer bindings after repeated treatment with CBD (5 mg/kg/day, subcutaneously [s.c.], for 16 days after the OA induction) vs. after the vehicle treatment, was also conducted.

**Figure 2:**
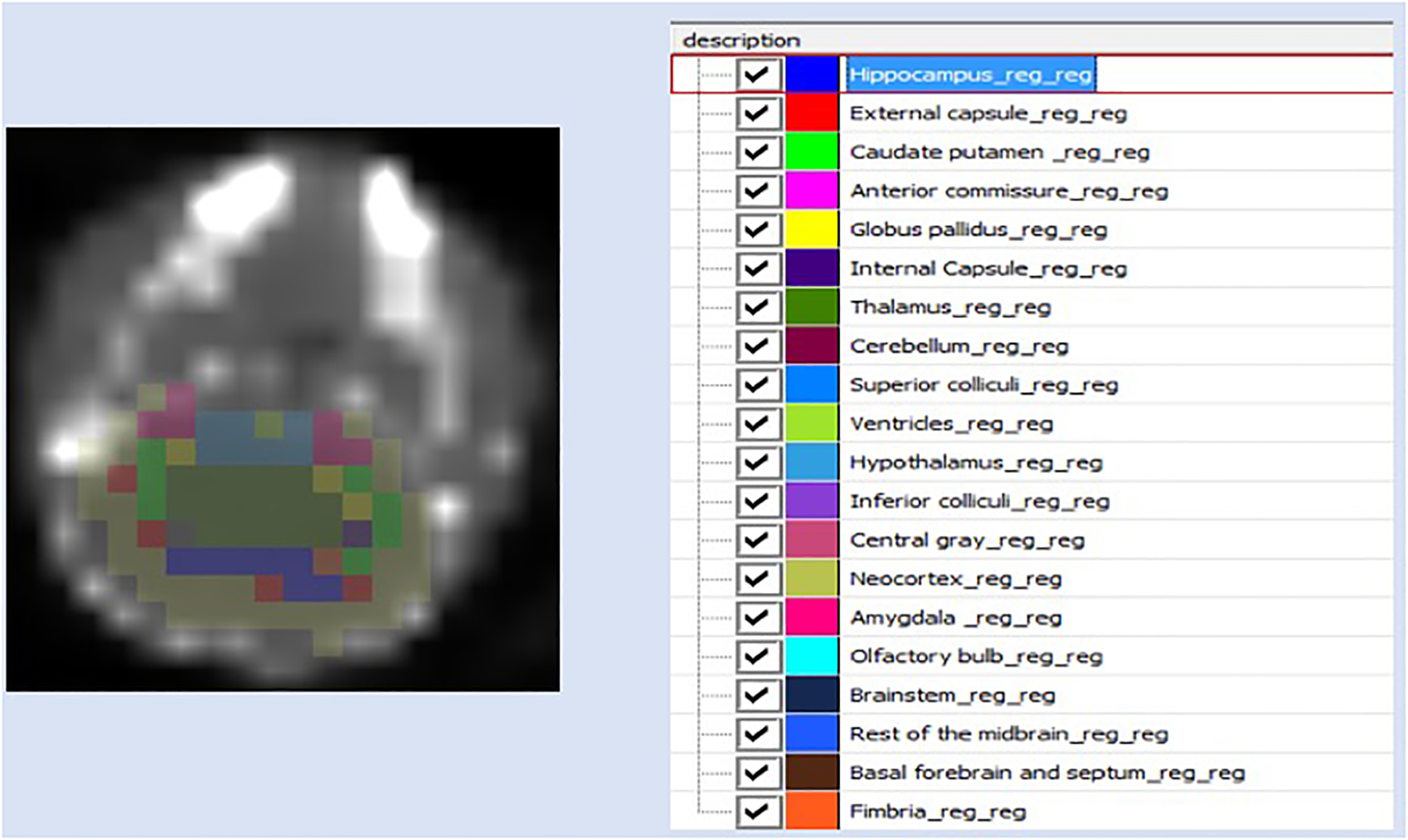
Brain segmentation to determine regional 5-HT_1A_ tracer binding

These study data were analyzed using the Acquisition Sinogram Image Processing (ASIPRO, Siemens) and Inveon Research Workplace (IRW, Siemens Medical Solutions USA, Inc.), and Firevoxel (wp.nyu.edu/Firevoxel) with an automated atlas-based brain mapping methodology that we have previously developed.(Mikheev et al., 2016) A 3D digital magnetic resonance microscopy (MRM)-based volume of interest (VOI) atlas generated from live C57BL/6J adult mouse brain was used for brain mapping and co-registration.(Ma et al., 2008) Briefly, based on our previously developed landmark-based co-registration, five landmarks (L/R mid ear, L/R eyes, and olfactory bulb) were drawn on the CT image of each subject. Single step co-registration of atlas (with the same five landmarks), CT and PET can be accomplished using Firevoxel, and the time-activity curves for the voxel intensity of specific regions of interest (ROI or VOI) of mouse brain were automatically generated after co-registration. Regional standard uptake values (SUV) were calculated as SUV = activity concentration (kBq/cc) in region of interest/[injected dose (MBq)/body weight (kg)]. SUV values of up to 20 brain regions can thus be obtained, including amygdala, brainstem, basal forebrain and septum, hippocampus, hypothalamus, olfactory bulb, cerebellum, etc.(Ma et al., 2008) (Figure 2) Cerebellum (CB) was chosen as a reference region (lowest density of 5HT_1A_ receptors), (Hillmer et al., 2014a; Hillmer et al., 2014b; Choi et al., 2015b; a) the corresponding averaged SUVR values (ratio of SUV(ROI) / SUV (CB)) of ROIs for last 10 minutes were compared in control animals, 13–20 days after OA induction (d13 or d20), and 16 days after treatment with either CBD or vehicle. For each time point, imaging studies were carried out in at least 6 animals (≥ 3 M and ≥ 3 F). Averaged SUVR values of 17 brain ROIs were used as the index for averaged brain 5-HT_1A_ availability (5-HT_1A_ functional activity), which were used for inter- and intra-subject comparisons across baseline-post-surgery-post-drug time points. Group comparisons among various time points for regional brain 5-HT_1A_ availabilities (SUVR values for last 10 min for male mice, and SUVR values for last 20 min for female mice to compensate for noisy data due to lower tracer uptake as result of lower 5-HT_1A_ receptors) were based on averaged SUVR values of all imaged animals, except for representative images displayed in Figures, which were derived from individual animals. For comparison purposes, comparative PET images are displayed as SUV images (SUV_25–30min_, kBq/cc) for individual animals [derived from the normalized SUV_max_ values based on the SUV(CB) intensity values of individual animals and scaled to the same appropriate SUVR scale ranges for all studies].

### 2.5. Behavioral Studies

All behavioral tests, including sensitivity to mechanical stimuli and behavioral measure of anxiety and depression, were carried out in the NYU Rodent Behavior Laboratory.

#### 2.5.1 Mechanical allodynia (von Frey tests):

Both electronic Von Frey assessment as well as the classic monofilament staircase method were conducted. Mechanical allodynia was assessed using monofilament/tips (0.02–1.6 g) of a logarithmic series von Frey fibers (Stoelting, Wood Dale, IL) and an electronic anesthesiometer (IITC). The threshold was captured as the lowest force (g) that evoked a rapid withdrawal response to 1 of 5 repetitive stimuli.(Tal and Bennett, 1994; Decosterd and Woolf, 2000) The mean 50% paw withdrawal threshold (g) was calculated from the manual Von Frey (Chaplan et al., 1994) for each group using Noldus Ethovision XT (v13.5) software.(Christensen et al., 2020) Sensitivity to mechanical stimuli of both hind paws were assessed at baseline (i.e., starting as a control mouse), ~20 d after MIA injection, before and after drug treatment. Electronic Von Frey (Auto VF), 50% paw withdrawal threshold and final filament (manual) pain thresholds for left and right paws of each animal were generated and compared among groups across various stages.

#### 2.5.2. Light/Dark box tests (L/D tests):

This procedure provides an index for “anxiety-like behavior” based on the profile of %light preference and total locomotor activity and velocity/total distance travelled during a L/D test session. In this procedure, mice were brought to the testing room to acclimate 1h prior to testing. A constant white noise was set at 65dB using a generator (San Diego Instruments) to mask extraneous sounds. The light/dark box apparatus consisted of a box (40 × 40 × 40 cm) that was half enclosed by black, opaque Perspex walls and ceiling and the other half with transparent walls and no ceiling. An overhead lamp was adjusted to give ~200 lux at the floor of the light compartment. The two compartments were connected by a small (5 × 5 cm) door in the dividing wall. Individual mice were placed into the dark chamber and allowed to freely explore the apparatus for 5 min. The latency and number of entries into the light compartment, the number of transitions and the total time spent in the light compartment were automatically quantified across the session using Noldus Ethovision XT (v13.5) software.

#### 2.5.3. Forced swim tests (FST tests):

This procedure provides an index of “behavioral despair” based on the profile of mobility during a forced swim session.(Porsolt et al., 2001) In this procedure, mice were brought to the testing room to acclimate 1h prior to testing. A constant white noise is set at 65dB using a generator (San Diego Instruments) to mask extraneous sounds that might influence swim behavior. For testing, each mouse is gently placed into the testing cylinder (40 cm height, 20 cm diam, Stoelting) with water level at 25 cm and temperature adjusted to 23–24 °C for a single 6-min session. Two cameras (one overhead and one side facing at water level) were positioned to video record each swimming session. Noldus Ethovision XT software (v13.5) was used to automatically track and score for activity and immobility.(Bambico et al., 2007)

### 2.6. Acute drug treatment

CBD was graciously provided by RTI International (Research Triangle Park, NC) via NIDA Drug Supply Program. This synthetic CBD with high purity (chromatographic purity of 99.5 ± 0.01% by GC [Total Area Analysis]) was required for the quantitative measurements specifically designed for the proposed study.

To investigate the interaction of CBD with the serotonin 5-HT_1A_ receptor, tracer bindings of [^18^F]MeFWAY in control animals were compared at the baseline (injection with tracer only), or after pretreatment of either an acute dose of CBD (1.0 mg/kg, i.v.) or the specific 5-HT_1A_ antagonist WAY1006235 (0.3 mg/kg, i.v.), at 5 min prior to tracer injection. The maximum volume used for a single injection was 0.1 mL.

### 2.7. Repeated drug treatment

Repeated treatment with CBD (5 mg/kg, s.c.) or vehicle (saline) was administered daily for 16 days in all study animals, starting from day 20 after OA induction with MIA. The dose, route of administration, and treatment time for CBD used in this study were based on literature. That is, the repeated low-dose CBD (5 mg/kg/day, s.c.)(De Gregorio et al., 2019) was based on a previously calculated value derived from the lowest i.v. acute dose (0.10 mg/kg) able to produce a significant alteration in 5-HT neuronal activity, taking into account the pharmacokinetic properties of CBD: Cmax, Tmax, and T_1/2_.(Samara et al., 1988; Mechoulam et al., 2002; Huestis, 2005; Deiana et al., 2012) This regimen mimics that used by patients using CBD to treat chronic neuropathic pain and anxiety.(Crippa et al., 2004; Whiting et al., 2015)

### 2.8. Statistical analysis

Data were analyzed using GraphPad Prism version 9.0.1 (Graph-Pad Software). Wilcoxon signed rank test was used to assess the differences for paired samples (within subject comparison). Changes from baseline values post-surgery or post-drug for each animal were calculated and the mean percent changes (±SEM) were determined for each treatment group (n = 10, 5 F and 5 M). Group comparisons in terms of regional measures from *in vivo* imaging data were conducted using Mann-Whitney *U* tests. The Student t-test was used to compare sex/gender difference and difference across various time-points. Pearson’s correlation was used to analyze correlation of *in vivo* regional tracer uptakes from neuroimaging and various parameter measures from three behavioral tests. For the behavioral data, a mixed-effects analysis was used with time point as within-subjects factor and drug treatment as between-subjects factor. Sidak’s tests (GraphPad Prism) were performed on significant main effects of time point or time point x drug treatment interactions to control the familywise error rate for multiple comparisons. A two-tailed probability value of 0.05 was used as the significance level.

## 3. Results

### 3.1. Radiotracer production

The total radiosynthesis of [^18^F]MeFWAY(Saigal et al., 2006; Wooten et al., 2011; Choi et al., 2013; Saigal et al., 2013) was accomplished in 52 min with an overall radiochemical yield of 24.0 ± 2.9 % (n = 6) decay corrected. Radiochemical purity was >99% and chemical purity was >98% with a molar activity (specific activity) of approximately 4.87±1.1 Ci/μmol (180.2 ± 40.0 GBq/μmol) at the end of radiosynthesis.

### 3.2. Acute CBD administration decreases [^18^F]MeFWAY binding, consistent with the imaging results after pretreatment with a specific 5-HT_1A_ antagonist

In order to demonstrate that CBD binds to 5-HT_1A_ target, CBD (1.0 mg/kg, i.v) was used as a pretreatment dose to block the tracer binding. Using a data-analysis method that we previously developed for single-step co-registration of atlas, CT and PET, tracer bindings for up to 20 brain regions were identified and quantitated. Time-activity curves (TAC) of 17 ROIs were derived, from which averaged SUVR values for each ROI for the last 10 min were calculated using CB as the reference region. Tracer bindings of [^18^F]MeFWAY in animals were then compared at the baseline (injection with tracer only) and after pretreatment of CBD or a known 5-HT_1A_ - specific drug.

**Figure 3:**
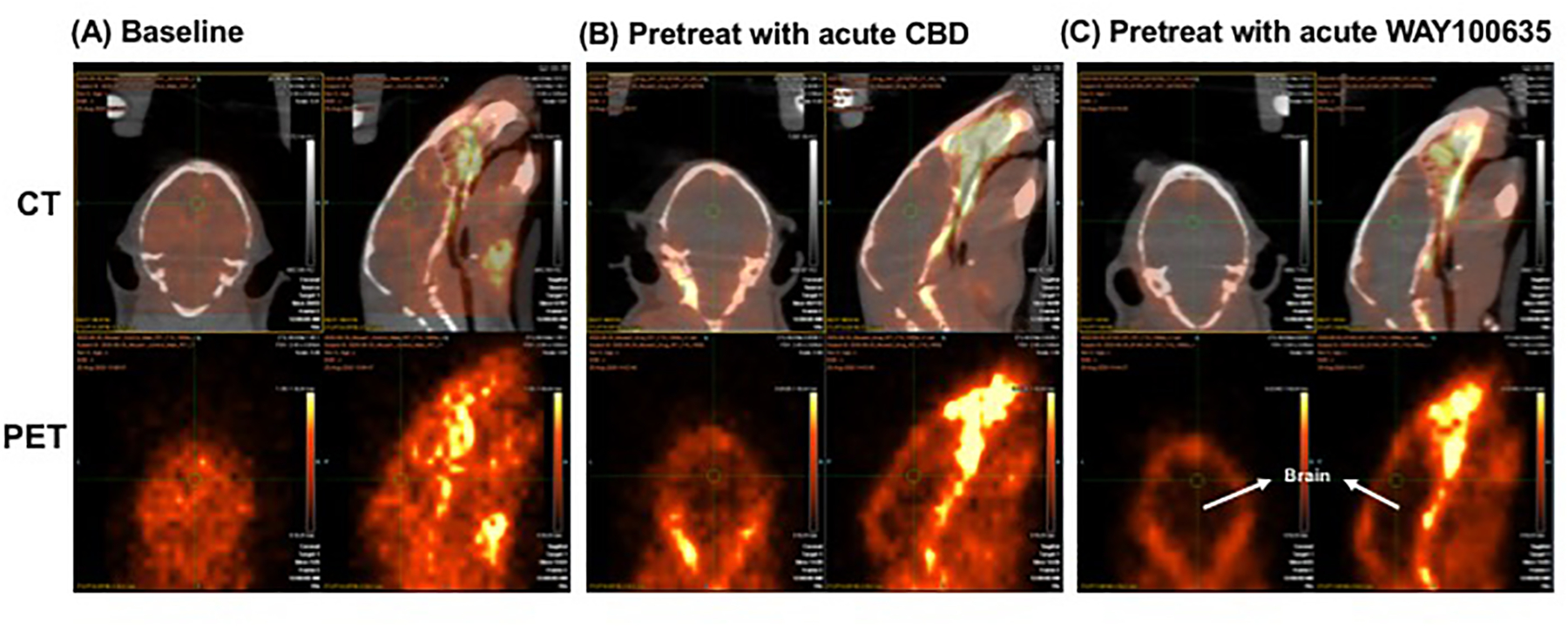
Representative images of [^18^F]MeFWAY studies in mice (coronal and sagittal views; top level displayed CT and PET co-registered images and bottom level displayed PET images alone): (A) baseline (injection with tracer only); (B) pretreatment with acute CBD (1 mg/kg, iv.); (C) pretreatment with acute WAY100635 (0.3 mg/kg, i.v.). The brain area, as indicated with arrows in both coronal and sagittal views in panel C, is the area delineated as the “black” area due to the significant reduction of tracer binding after pretreatment with WAY100635. As seen, there was a significant reduction of tracer binding in the brain area in panel B and C, as indicated by the black area with lower brain uptake of the radiotracer when compared with the corresponding brain area in panel A of the baseline study. For comparison purposes, comparative PET images were displayed as SUV images (SUV_25–30_, kBq/cc) for individual animals [derived from the normalized SUV_max_ values based on the SUV(CB) intensity values of individual animals and scaled to the same SUVR scale range (0–6.5) for all studies].

As shown in representative images (Figure 3), tracer binding was significantly reduced in animals after acute doses of CBD (1.0 mg/kg, i.v.) (panel B), as compared to that for baseline (panel A). This binding specificity of CBD to 5-HT_1A_ was further confirmed by a similar reduction of tracer binding when the specific 5-HT_1A_ antagonist WAY1006235 was used (0.3 mg/kg, i.v., 5 min prior) (panel C). That is, the brain area, as indicated with arrows in both coronal and sagittal views in panel C, is the area delineated as the “black” area due to the significant reduction of tracer binding after pretreatment with the selective 5-HT_1A_ blocker (WAY100635). As shown, there was a significant reduction of tracer binding in the brain area in panel C and B, as indicated by the black area with lower brain uptake of the radiotracer when compared with the corresponding brain area in panel A of the baseline study. The observed higher uptake outside the brain area in panel B or C may be due to (a) decreased binding sites in the brain (resulting from the occupancy of 5-HT_1A_ receptors by the blockers); (b) increased amount of metabolites (resulting from increased metabolism of the radiotracer) that could not cross the blood-brain barrier; and/or (c) free [^18^F]fluoride (resulting from defluorination of the radiotracer and its metabolites) that trapped in skull bones surrounding the brain area. Some of these events may occur even for baseline study after injection of the radiotracer alone as shown in Panel A with a few bright spots outside the brain; however, these events would be exacerbated after pretreatment with the blockers.

**Figure 4:**
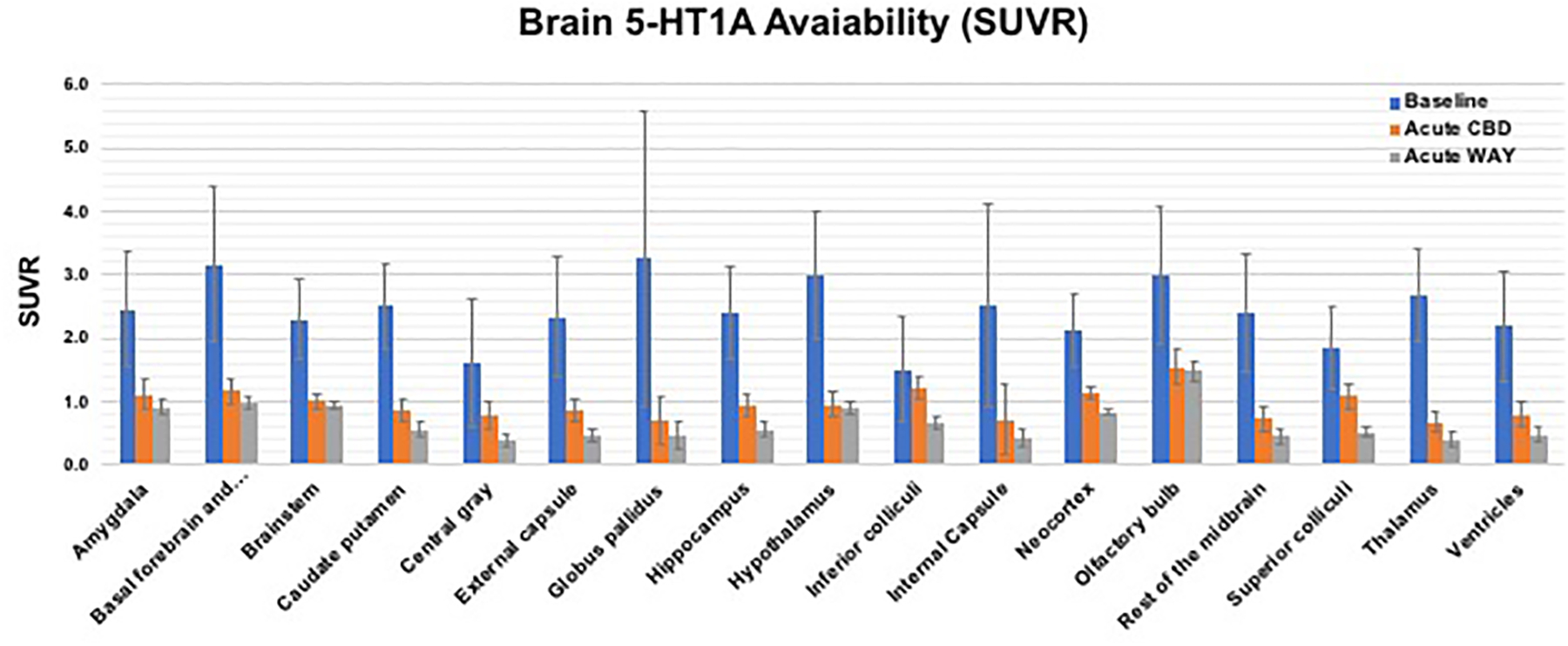
Bar graphs of averaged regional brain 5-HT_1A_ availability (SUVR values for the last 10 min calculated using cerebellum as the reference region) at baseline (blue bar), and after pretreatment with either CBD (orange) or WAY100635 (grey). (n = 3–4 each group)

As shown in Figure 4, tracer bindings (SUVR values) for all 17 brain ROIs were highest for baseline (blue bars), significantly reduced after CBD pretreatment (orange bar), and lowest bindings after pretreatment with a specific 5-HT_1A_ antagonist (WAY100635, 0.3 mg/kg, i.v., grey bar). A quantitative measurement using the averaged SUVR values of 17 brain ROIs: 2.43 ± 0.49 (baseline), 0.96 ± 0.23 (after acute CBD, with 60% reduction), and 0.68 ± 0.29 (after acute WAY100635, with 72% reduction), suggest the specific binding of CBD to the 5-HT_1A_ target. These averaged SUVR values of 17 brain ROIs were then used as the index for averaged brain 5-HT_1A_ availability (i.e., global 5-HT_1A_ functional activity in the brain) to compare the subsequent tracer bindings after OA induction and after drug treatment.

### 3.3. Reduction of 5-HT_1A_ activity/availability after OA induction can be restored by repeated low-dose CBD treatment, with no significant improvement after vehicle treatment

After baseline tracer bindings in control animals were established (via both PET/CT neuroimaging and behavioral tests), the same animals were subject to MIA injections (1 mg of MIA in 10 μL saline) to induce OA, according to the paradigm described in the Method section. The same PET/CT imaging and behavioral testing protocols were performed on day 13 and/or day 20 post-MIA injection. Daily low-dose CBD treatment (5 mg/kg/day, s.c.) on half of the OA animals started at day 20 post-MIA injection, while the other half received vehicle treatment. The same imaging and behavioral testing protocols were again performed after the drug treatment. With this sequential study design, direct assessment in terms of neurochemical and behavioral changes in the same animals at different stages [i.e., baseline, after OA induction, and after drug treatment] or in different groups (CBD vs. vehicle) can thus be compared.

**Figure 5:**
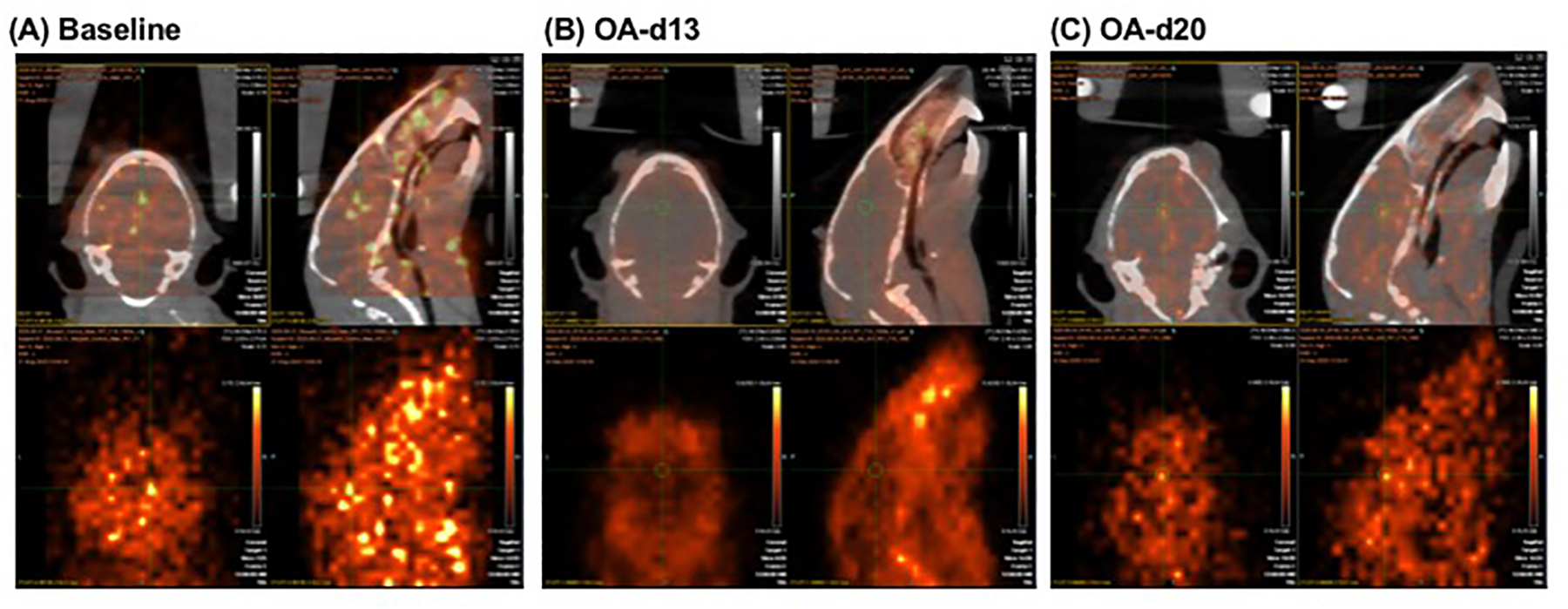
Representative images (coronal and sagittal views) to indicate changes of 5-HT_1A_ tracer binding at d13 (B) and d20 (C) after the OA induction, as compared to baseline (A). A larger reduction in tracer binding was seen in day 13 animals. For comparison purposes, comparative PET images were displayed as SUV images (SUV_25–30min_, kBq/cc) for individual animals [derived from the normalized SUV_max_ values based on the SUV(CB) intensity values of individual animals and scaled to the same SUVR scale range (0–4) for all studies].

Averaged SUVR values derived from 17 ROIs (using CB as the reference region) as index for brain 5-HT_1A_ activities and availability were used for tracer binding evaluation and comparison at different stages and between groups. The tracer binding after OA induction (via MIA injection) was significantly reduced as compared to baseline binding in control animals (particularly in male mice as the data describe here). The decreased bindings were computed for the post-surgery animals with avg. SUVR of 1.13 ± 0.31 and 1.45 ± 0.29 (from 17 ROIs) for day 13 and day 20, respectively, which indicated an avg. reduction binding of 45% and 28% from baseline binding (SUVR 2.00 ± 0.31). Representative images for d13 and d20, as compared to baseline, are shown in Figure 5. These results are consistent with the notion that 5-HT_1A_ plays a role in the modulation of pain and anxiety. Further, these results are consistent with results from previous human studies indicating that chronic pain was associated with low 5-HT_1A_ receptor.(Lindstedt et al., 2011),(Drevets et al., 2007; Sato et al., 2008) It was also noticed that the decreased tracer bindings appeared to be larger in some animals at day 13 as compared to day 20.

**Figure 6:**
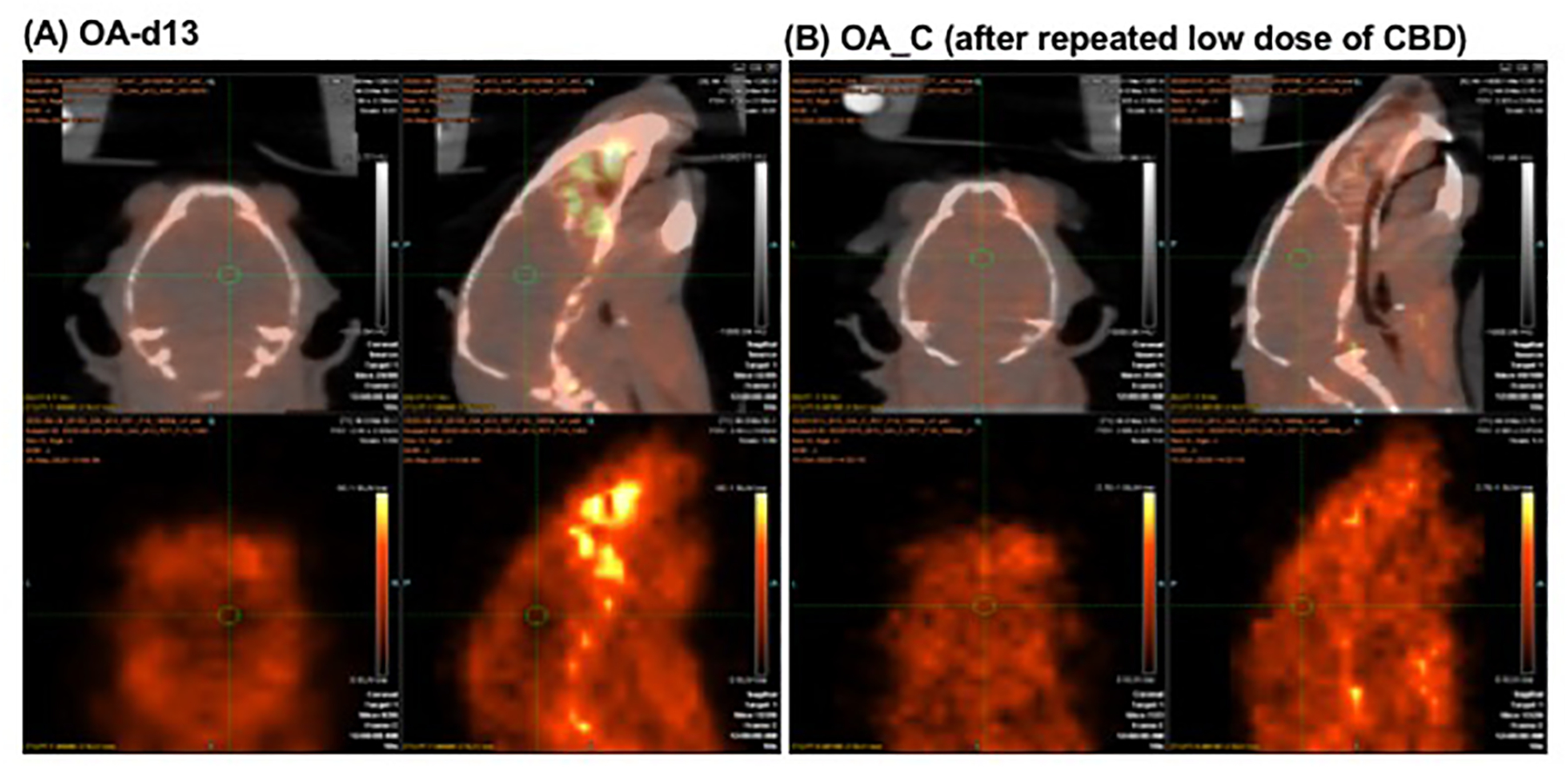
Representative images (coronal and sagittal views) to indicate changes of 5-HT_1A_ tracer binding at d13 after the OA induction (A) vs. an increased tracer binding after repeated low-dose of CBD treatment (5 mg/kg, s.c.) for 16 days (B). For comparison purposes, comparative PET images were displayed as SUV images (SUV_25–30min_, kBq/cc) for individual animals [derived from the normalized SUV_max_ values based on the SUV(CB) intensity values of individual animals and scaled to the same SUVR scale range (0–4) for all studies].

**Figure 7:**
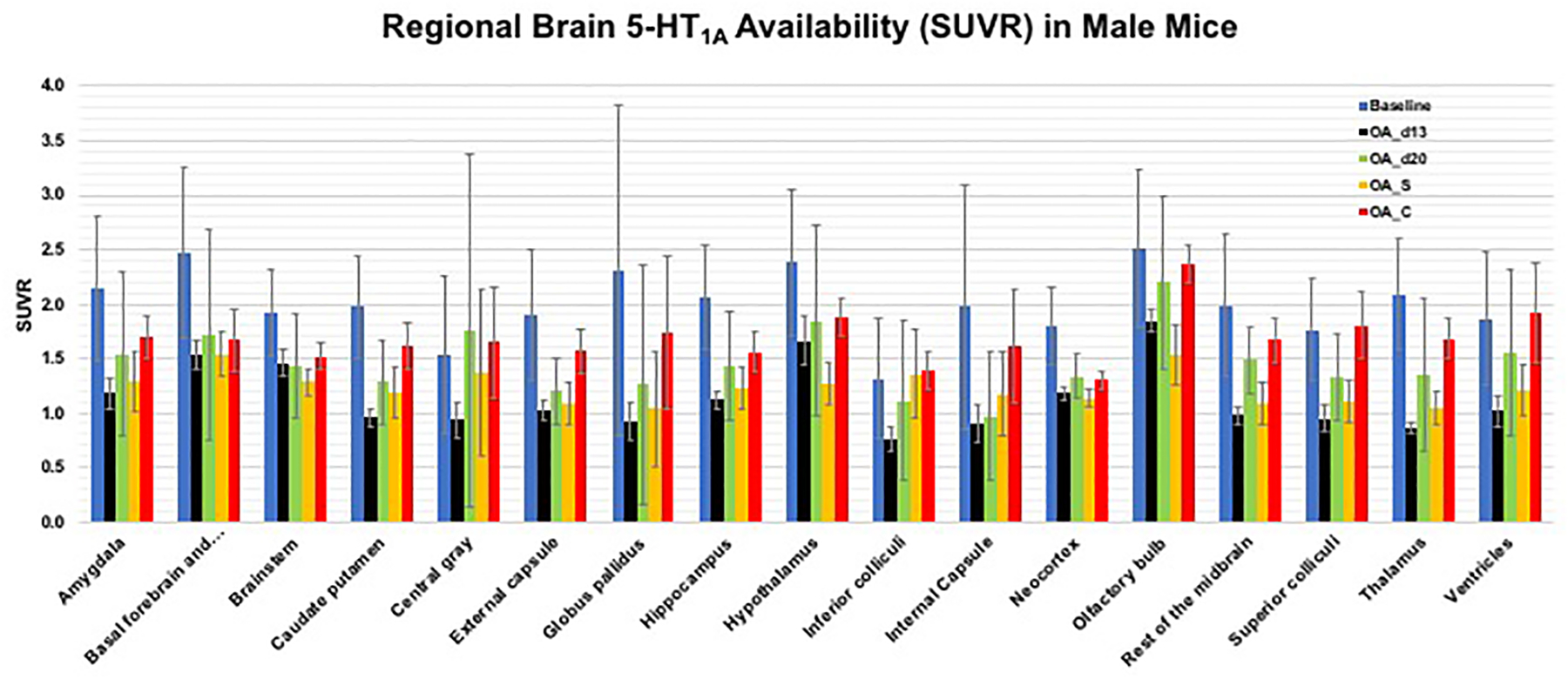
Bar graphs to indicate averaged regional changes in 5-HT_1A_ tracer binding (SUVR values for the last 10 min calculated using cerebellum as the reference region) of 17 ROIs for male mice across various time points; Baseline, OA_d13, OA_d20, OA_S and OA_C [OA_d13 and OA_d20 are groups of mice post-injection of MIA at day 13 and day 20, respectively. OA_S and OA_C are groups of mice treated for 16 days with CBD (5 mg/kg, s.c.) and saline, respectively.] (n = 3–4 for each group)

**Figure 8:**
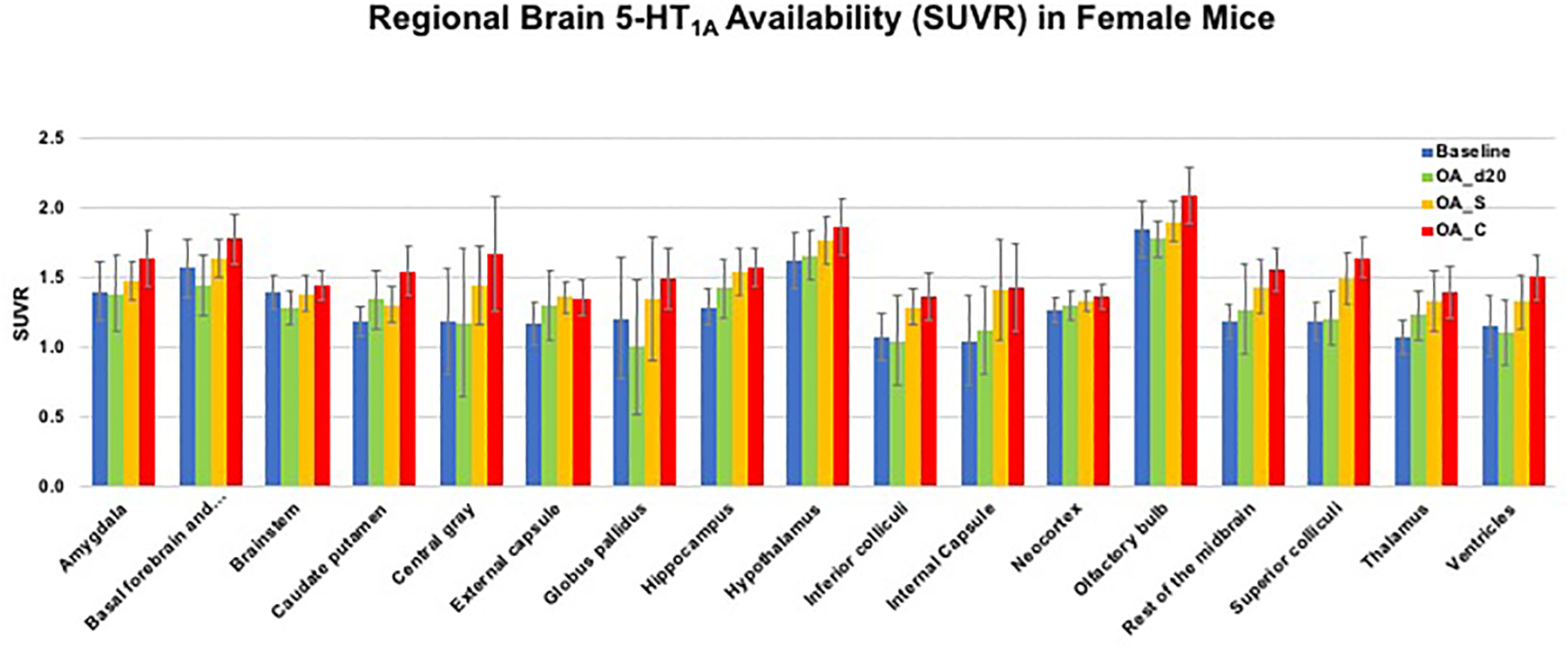
Bar graphs to indicate averaged regional changes in 5-HT_1A_ tracer binding (SUVR values for the last 20 min calculated using cerebellum as the reference region) of 17 ROIs for female mice across various time points; Baseline, OA_d20, OA_S and OA_C [OA_d20 are groups of mice post-injection of MIA at day 20. OA_S and OA_C are groups of mice treated for 16 days with CBD (5 mg/kg, s.c.) and saline, respectively.] (n = 3–4 for each group)

After repeated low-dose CBD treatment (5 mg/kg/day, s.c.) for 16 days, the tracer bindings were increased and approached to the baseline binding level (1.69 ± 0.23), while no *improvement was observed after vehicle treatment (*1.23 ± 0.15). The treatment difference reached significance *(p* < 0.001) and representative images for OA_d13 vs. after CBD treatment are shown in Figure 6. Brain 5-HT_1A_ availability for 17 ROIs are plotted out to indicate the changes at various stages (baseline, OA_d13, OA_d20, OA_S and OA_C) as shown in Figure 7 (for male mice). Bar graphs to indicate averaged regional changes in 5-HT_1A_ tracer binding for female mice across various time points are presented in Figure 8 and discussed below in the Sex differences section. These results suggest that CBD can be used as a potential drug for treatment of OA.

### 3.4. Behavioral Assessment

A group of mice were induced OA via MIA injection (1 mg of MIA in 10 μL saline) into the right knee, and after 20 days from the surgery, half of the OA animals were treated with CBD (5 mg/kg/day, s.c.) and the other half OA animals received vehicle treatment, for 16 days. The same behavioral testing protocols (von Frey, Light/Dark Box, and Forced Swim Test) were tested on all animals at baseline, day 20 after OA induction, and after 16 days of drug treatment.

#### Von Frey Assessment:

**Figure 9:**
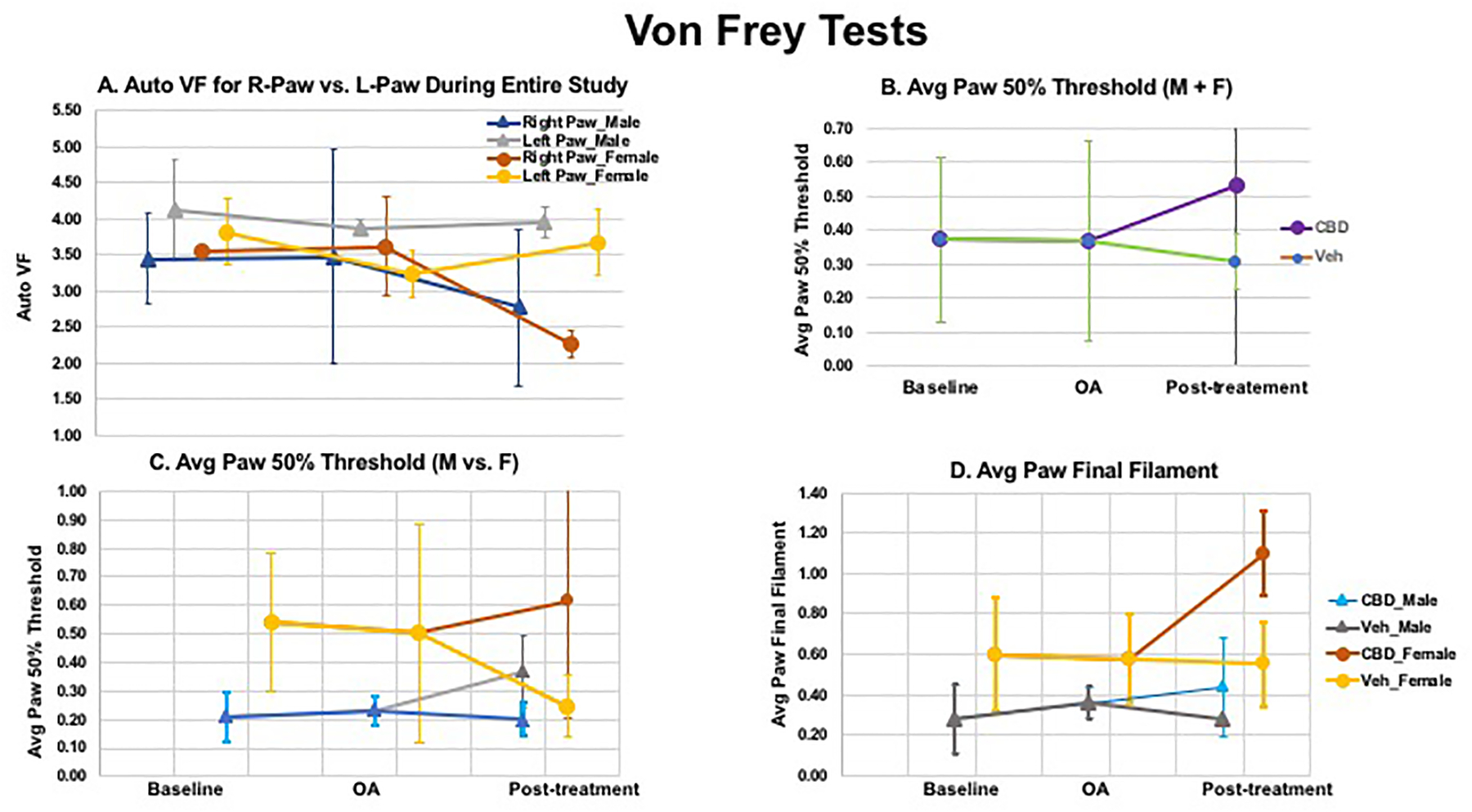
Von Frey tests: A. Comparison of R-paw vs. L-paw during the entire study: values for electronic Von Frey (Auto VF) withdraw threshold (g) for R-paw (MIA injected) vs. L-paw (vehicle injected) of male and female mice were compared. A progressive increase in mechanical sensitivity (decrease pain threshold) of the right hind paw following OA surgery, with no significant changes in sensitivity in the left hind paw, was observed. Figures 9B–9D are presented by using averaged paw (L and R) and pooling all data from baseline and OA (under the same paradigm) and separate them based on the different drug treatment. Plots (avg paw 50% withdrawal) are presented when both sex are included (B) and separately (C), to clearly indicate the sex/gender difference. The averaged paw 50% threshold measurement appeared to be decoupled in males as compared to females (CBD vs. vehicle), but not with the averaged paw filament measurement (Figure D). (data were derived from 5 M and 5 F).

The pattern observed in the Von Frey assessment across baseline—post-surgery—post-injection time points was a decrease in mechanosensory pain threshold (increase in pain sensitivity) only in the later, post-treatment period. The decrease was primarily driven by increases in sensitivity of the right hind limb (right knee joint was the MIA injection site). As shown in Figure 9A, comparison between R-paw (MIA injected) and L-paw (vehicle injected) throughout the entire period of the study (without CBD treatment) for both male and female mice, a progressive increase in mechanical sensitivity (decrease pain threshold) of the right hind paw following OA surgery, with no significant changes in sensitivity in the left hind paw, was observed. However, the pain thresholds at the baseline and early postsurgical Von Frey time points were largely similar (though the extent of the decreases in pain thresholds was considerably lower than those in our previous pilot studies (unpublished)). These results were significant only for electronic Von Frey assessment (Auto VF, Figure 9A), but showed a similar trend for the classic monofilament staircase method. Overall, this pattern suggests that ipsilateral pain sensitivity shows a delayed peak increase after OA-MIA knee surgery, consistent with the prior literature.

This delayed pain sensitivity increases post-surgery and into the post-drug injection period, particularly in the ipsilateral (right) paw, may have complicated the interpretation of the study results (i.e., the pain-threshold changes might result from either OA-induction or drug treatment, or both). Further, the compensatory patterns shared between left and right paws were observed in our studies.(Lakes and Allen, 2016) Thus, Figures 9B–9D are presented by using averaged paw (L and R) and pooling all data from baseline and OA (under the same paradigm) and separate them based on the different drug treatment. Plots (avg. paw 50% withdrawal) are presented when both sex are included (**B)** and separately **(C)**, to clearly indicate the sex/gender difference. The averaged paw 50% threshold measurement appeared to be decoupled in males as compared to females (CBD vs. vehicle), but not with filament measurement (Figure 9D).

#### Light/Dark Box:

**Figure 10:**
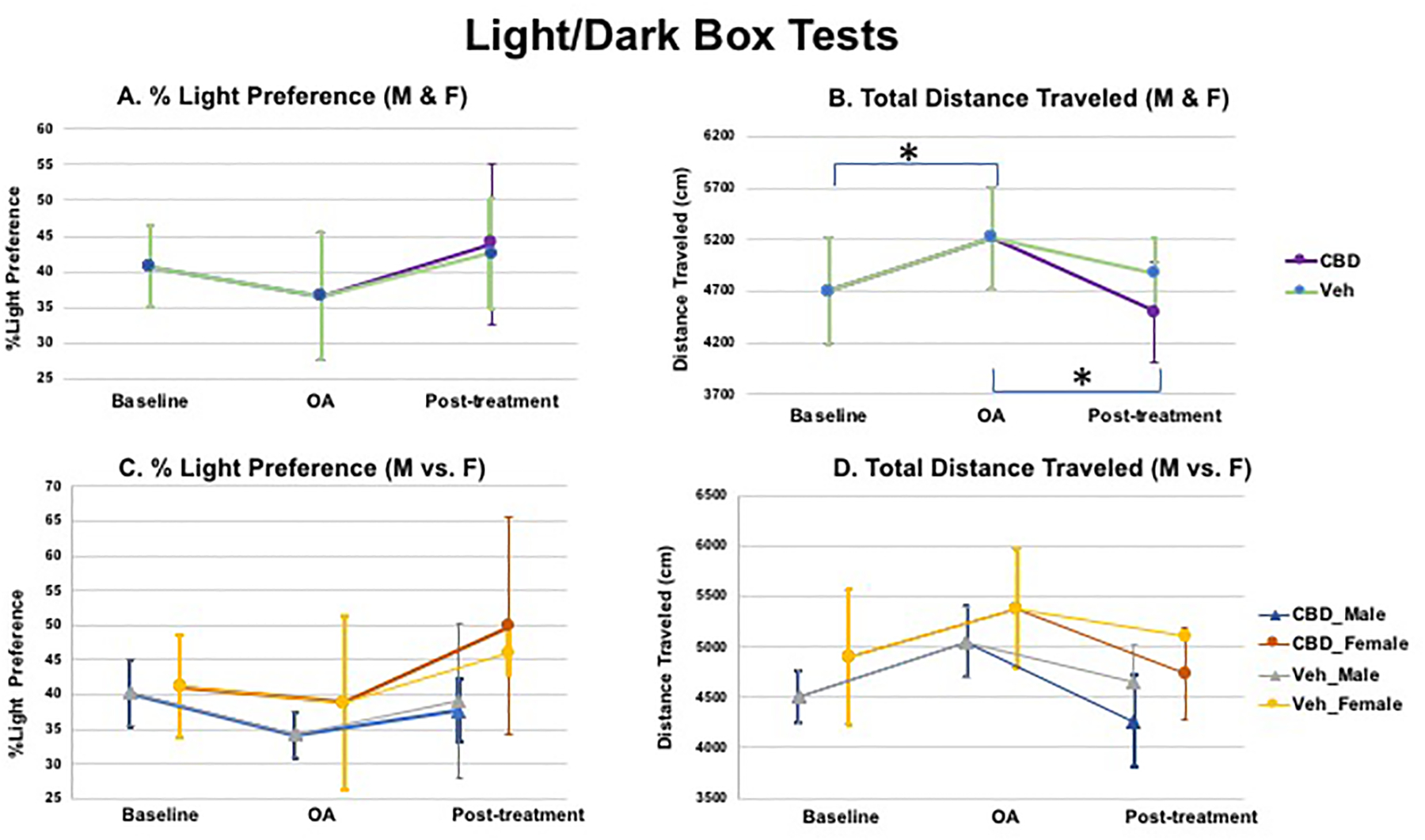
Light/Dark box tests: A (%light preference) & B (total distance traveled) when both males and females were included (top panel); C (%light preference) & D (total distance traveled) when males and females are separated in data analysis (data were derived from 5 M and 5 F). Less preference to light (A & C; did not reach significance) or increased distance traveled (B & D; *p < 0.05) were observed for both male and female mice post-OA surgery, as compared to baseline, suggesting a potential increase in anxiety after OA induction. The extent of decreased distance traveled (reduction of anxiety) was higher after CBD treatment than that after vehicle treatment. Females appeared to exhibit less anxiety-like behaviors (less reduction of %light preference) after OA induction, and were more sensitive to the CBD treatment, as compared to males (C & D).

The L/D box test assesses the innate aversive behavior of rodents to bright areas as well as their stress induced-natural exploratory response. In general, spending more time in the dark compartment (i.e., decrease % light preference) and increasing velocity/total distance traveled are signs of increased stress-induced anxiety-like behavior. The profile across the 3 time periods (baseline-post-surgery-post-drug) in the Light/Dark box is U-shaped for % light preference, and inverted-U shaped for total locomotor activity and velocity/total distance travelled (Figure 10A & 10B). These patterns suggest higher anxiety (e.g., dark preference and escape or mild stress-like locomotor activity) at the post-surgery time point (OA stage), which recovers back toward baseline levels after the daily drug injection period.

The difference for locomotor activity and velocity/total distance traveled between baseline and OA, and between OA and after CBD treatment reached significance (*p* < 0.05, Figure 10B), but the difference was not significant between OA and after vehicle treatment. There was only a very strong trend for %light preference; however, the difference did not reach significance (Figure 10A).

#### Forced Swim Test:

**Figure 11:**
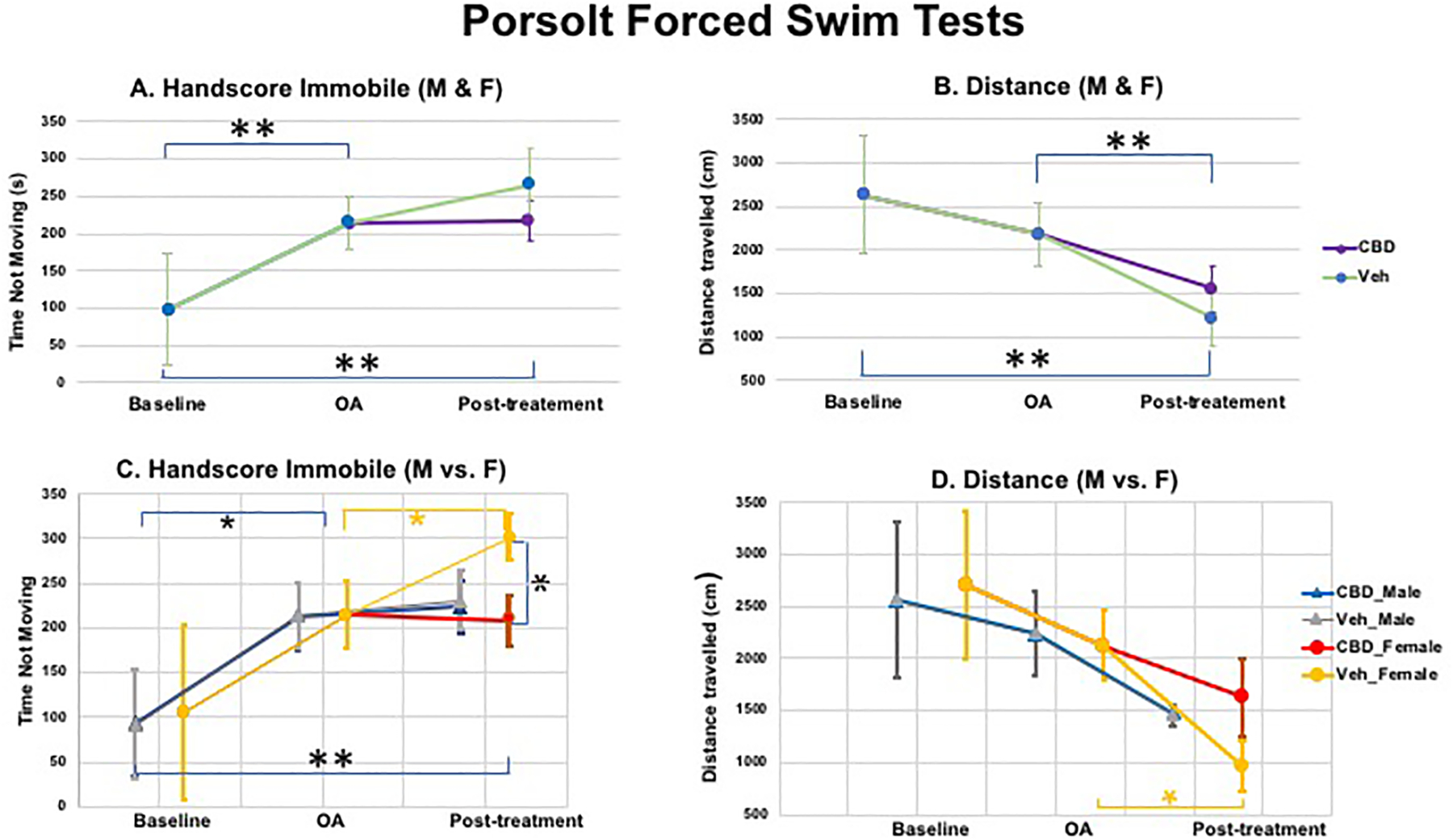
Forced Swim tests: A (immobility) & B (total distance traveled) when both males and females were included (top panel); C (immobility) & D (total distance traveled) when males and females are separated in data analysis (data were derived from 5 M and 5 F; *denoted for *p* < 0.05, and ** denoted for *p* < 0.005). When both M & F were included, immobility in FST showed significantly different (**p < 0.005) between baseline vs. OA, and baseline vs. post-treatment, while distance traveled showed significantly different (**p < 0.005) between baseline vs. post-treatment, and OA vs. post-treatment. In M vs. F separate analysis, male mice showed significant difference in immobility between baseline and OA; while female mice showed significant immobility differences among baseline vs. OA, OA vs. Veh, and CBD vs. Veh.

The profile in the Porsolt forced swim test was a monotonic decrease in *locomotor activity* and a monotonic increase in *time not moving* (immobility) across the 3 time periods (baseline—post-surgery—post-drug). These two measures are highly correlated (R^2^ > 0.8) with negative Pearson correlation *r* values of −0.92, −0.92 and −0.64, respectively, suggesting that they may jointly reflect depressive-like behaviors (passive coping behaviors). The differences in depressive-like behaviors (e.g., for total distance traveled) reached significance across the 3 time periods (Figure 11A and 11B, *denoted for *p* < 0.05 and **denoted for *p* < 0.005), though not significantly different for immobility between post-surgery and post-drug period (Figure 11A). These results suggest that depressive-like behavior increased progressively across each time point, with a slightly larger increase in depressive-like behavior (e.g., steeper slope) at the later post-drug time point. Von Frey pain sensitivity is moderately associated with Porsolt measures at baseline and post-drug injection periods (r ~ 0.45), which was expected based on the known links between pain and depressive-like behaviors. Interestingly, however, mechanosensory pain appears decoupled from swim test measures in the post-surgery period (r ~ 0). This is consistent with the observed increase in stress and anxiety-like behavior at the post-surgery time point, despite no consistent increase in pain sensitivity ─ suggesting that stress/inflammation may be impacting pain assessment (e.g., hypoalgesia effects of stress/inflammation in some mice).

Light/Dark box (L/D) measures were strongly associated with Porsolt measures at baseline (r ~ 0.79), and moderately associated at post-surgery and post-drug injection periods (r ~ 0.39). %Light preference measured in L/D box was strongly correlated with von Frey indices of pain in the post-surgical time period (*r* ~ 0.65), consistent with the notion that higher post-surgical anxiety may relate to OA. However, locomotor activity was not strongly correlated with von Frey indices of pain in the post-surgical time period (either paw – R^2^ ~ 0.04), suggesting that pain may not be simply related to locomotor activity of anxiety in the light/dark paradigm. There is a moderately high correlation (R^2^ < 0.5) between locomotor activity and preference for the bright compartment in the post drug injection period, both of which are slightly related to pain threshold (r = 0.37 - higher pain threshold associated with higher activity and lower anxiety).

There were sex differences in pain perception/expression and in response to drug treatment (CBD vs. vehicle) (see descriptions below).

### 3.5. Sex differences

The general trend for both neurochemical and behavioral changes across the 3 time periods is similar for all mice as described above. In subgroup analyses, sex differences between male and female mice were revealed. The details are described below.

#### Sex differences in neurochemical changes measured with in vivo PET:

The neuroimaging data presented above were mostly derived from males (Figures 3–7). That is, the differences in tracer bindings reached significance for males across various time periods (baseline, OA_d13, OA_d20, OA-S, and OA_C) (Figure 7).

According to our imaging studies, female mice displayed overall significantly lower averaged 5-HT_1A_ availability (lower 5-HT_1A_ SUVR values for most brain ROIs) as compared to male mice (*p* < 0.001, n = 4 each).

As shown in Figure 8, the difference in tracer binding for females between baseline and after OA reduction did not reach significance. However, after repeated low-dose CBD treatment for 16 days, the averaged tracer bindings were significantly increased (*p* <0.001) and reached to the baseline binding level, while no significant improvement was observed with vehicle treatment (after adjustment with recovery days; i.e., 36 days post OA induction with MIA injection). The tracer binding differences (global 5-HT_1A_ functional activity in the brain) between CBD vs. vehicle (saline) treatment reached significance for both males and females.

#### Sex differences in behavioral measures:

The behavioral assessment via various tests appeared to correlate quite well with neurochemical changes measured with PET. The details are described below.

For Von Frey tests, the results obtained suggested a potential sex difference in pain sensitivity and responses. The results were compared using either electronic Von Frey for averaged 50% paw withdrawal threshold or classic monofilament staircase method, with and without sex separation. As seen in Figure 9A (paw 50% withdrawal) when both males and females were included, the pain thresholds at the baseline and early postsurgical Von Frey time points were largely similar (similar results for paw filament measures, not shown). A decrease in mechanosensory pain threshold after OA surgery (i.e., increase in pain sensitivity) was observed only in the later, post-injection period. However, an increased pain threshold (reduction of pain) after repeated low-dose CBD treatment and no improvement after vehicle treatment were observed. On the other hand, when males and females are separated in data analysis as seen in Figure 9B (paw 50% withdrawal) **& 9C** (paw filament), the results suggested that females appeared to be less responsive at the baseline towards pain stimuli; however, they appeared more sensitive to pain perception after OA induction, and to the CBD treatment. The paw 50% threshold measurement appeared to be decoupled in male (CBD vs. vehicle, Figure 9B), but not with filament measurement (Figure 9C).

Light/Dark box tests on %light preference and total distance traveled are shown in Figure 10. As seen in Figure 10A & 10B (top panel, when both males and females were included) or in 10C & 10D (bottom panel, when males and females are separated in data analysis; data were derived from 5 M and 5 F), less preference to light (**A & C**, did not reach significance) or increased distance traveled (**B & D**; *p < 0.05) were observed for both male and female mice post-OA surgery, as compared to baseline, suggesting a potential increase in anxiety after OA induction. The extent of decreased distance traveled (reduction of anxiety) was higher after CBD treatment than that after vehicle treatment. Further, females exhibited less anxiety-like behaviors (less reduction of %light preference) after OA induction, and were more sensitive to the CBD treatment, as compared to males (**C & D**).

For Porsolt-Forced Swim tests, as seen in Figure 11 A & B (when both males and females were included) or in Figure 11 C & D (when males and females are separated in data analysis), increased immobility (**A** & **C**) or decreased distance traveled (**B** & **D**) for both male and female mice post-OA surgery was observed, as compared to baseline, suggesting a potential increase in depression after OA induction (data were derived from 5 M and 5 F; *denoted for *p* < 0.05, and ** denoted for *p* < 0.005). Further, females appeared to be more sensitive to CBD treatment (i.e., decreased depressive-like behaviors, *p* < 0.05), as compared to males (no significant difference between CBD treatment and vehicle treatment, Figure 11 C & D).

Based on these preliminary results, there appeared to be sex differences between male and female mice in the model of OA. Specifically, female mice have lower 5-HT_1A_ availability and are less responsive to pain stimuli at the baseline, as compared to male mice. Females appeared to have less neurochemical change (i.e., less 5-HT_1A_ tracer binding change) and less change in anxiety-like behavior (less reduction of %light preference) than male mice after OA induction. However, females appear to be more sensitive to CBD treatment with significantly increased 5-HT_1A_ binding (OA_d20 vs. OA_C, *p* < 0.001, Figure 8), and higher degree of restoration in anxiety-like or depressive-like behaviors after CBD treatment, particularly for females (Figure 10 and 11).

## 4. Discussion

A major symptom of patients with OA is pain that is triggered by peripheral as well as central changes within the pain pathways. The current treatments for OA pain such as NSAIDS or opiates are neither sufficiently effective nor devoid of detrimental side effects. Animal models of OA are being developed to improve our understanding of OA-related pain mechanisms and define novel pharmacological targets for therapy. Currently available models of OA in rodents include surgical and chemical interventions into one knee joint. The MIA-induced OA model has become a standard for modeling joint disruption in OA in both rats and mice. The model, which is easier to perform in rodents, involves injection of MIA into a knee joint, which induces rapid pain-like responses in the ipsilateral limb, the level of which can be controlled by injection of different doses. Intra-articular injection of MIA disrupts chondrocyte glycolysis by inhibiting glyceraldehyde-3-phosphatase dehydrogenase and results in chondrocyte death, neovascularization, subchondral bone necrosis and collapse, as well as inflammation. The morphological changes of the articular cartilage and bone disruption are reflective of some aspects of patient pathology. Along with joint damage, MIA injection induces referred mechanical sensitivity in the ipsilateral hind paw and weight bearing deficits that are measurable and quantifiable. These behavioral changes resemble some of the symptoms reported by the patient population, thereby validating the MIA injection in the knee as a useful and relevant preclinical model of OA pain. Pain mechanisms of OA presumably involve peripheral and central sensitization, and inflammation.(Schuelert and McDougall, 2009; Parks et al., 2011; Havelin et al., 2016; Miller et al., 2017) The advantage of small-animal imaging with PET using mice is that its whole body can be in the field of view during PET imaging; thus, the sites of pain modulation (central and/or peripheral) can both be measured and correlated *via* PET for specific targets. Further, the pain threshold measures from behavioral tests after CBD can be linked with the PET imaging results.

The pain experienced by OA patients is defined as “mixed pain” since it is due to local inflammation, tissue degeneration, and alteration of the threshold for tactile stimuli set by the central nervous system.(Clauw and Hassett, 2017) This complex mechanism of pain sensitivity explains why pain of OA patients does not correlate with cartilage and bone structural changes.(Hannan et al., 2000) Little is known about the exact target engagement for the analgesic, anxiolytic, and anti-inflammatory effects of CBD in the brain. Our *in vivo* neuroimaging, combined with behavioral assessment, will permit us to link CBD modulation for *neurochemical* changes to its analgesic properties, as reflected in *behavioral* changes. With these multiple, synergistic, yet independent approaches, the mechanisms of CBD can be further confirmed.

Pain is often in comorbidity with mood and anxiety disorders in patients with OA.(Rosemann et al., 2007; Axford et al., 2010) 5-HT_1A_ receptor is the most abundant serotonin subtype expressed in the regions of brain such as prefrontal cortex, limbic system, and hypothalamus that receives serotonergic input from the raphe nuclei. CBD displays allosteric agonism on 5-HT_1A_ receptor and it has been suggested that CBD interacts with the serotonin 5-HT_1A_ receptor, which may result in CBD’s analgesic and anxiolytic effects.(Zuardi et al., 1982; Zuardi et al., 1991; Zuardi et al., 1993; Zuardi et al., 1995; Crippa et al., 2004; Moreira and Guimaraes, 2005; Zanelati et al., 2010; Bergamaschi et al., 2011; Linge et al., 2016) However, this link has neither been examined nor established in OA. In this first *in vivo* PET imaging study in mice, we investigated the interaction of CBD with serotonin 5-HT_1A_ receptor via a combination of *in vivo* neuroimaging and behavioral studies in a well-validated OA animal model across baseline—post surgery—post drug treatment time points.

Using the MIA-induced OA model, our PET imaging with the selective 5-HT_1A_ tracer [^18^F]MeFWAY clearly demonstrated, for the first time, the interaction of CBD with 5-HT_1A_ receptor, the target that is implicated in pain, depression, and anxiety. First, we showed that the tracer binding in control animals was significantly reduced after acute doses of CBD (1.0 mg/kg, i.v.). This binding specificity to 5-HT_1A_ was corroborated by a significant reduction of tracer binding when the specific 5-HT_1A_ antagonist WAY1006235 (0.3 mg/kg, i.v) was used. Further, previous human studies suggested that chronic pain is associated with low 5-HT_1A_ receptor.(Lindstedt et al., 2011) In addition, lower 5-HT_1A_ receptor densities were found in depressed rats(Sato et al., 2008) as well as depressed patients.(Drevets et al., 2007) Our results of decreased 5-HT_1A_ tracer binding in OA animals (i.e., decreased 5-HT_1A_ *in vivo* availability) are consistent with these previous studies.(Drevets et al., 2007; Sato et al., 2008; Lindstedt et al., 2011) Most importantly, we also showed that repeated treatment with CBD was able to reverse the MIA-induced deficits in brain 5-HT_1A_ neuronal activity, while no significant improvement was observed after vehicle treatment. That is, via *in vivo* imaging strategies we demonstrated the regional 5-HT_1A_ availability changes in the brain, as indicated via averaged SUVR values (of 17 ROIs), and they reached significance across various time points, as shown in Figure 7.

These results derived from our neuroimaging studies, including blocking studies with an acute dose of CBD in control animals and restoration of the MIA-induced deficits in brain 5-HT_1A_ neuronal activity after repeated treatment with CBD, suggest that CBD acts directly on 5-HT_1A_. Other possible mechanisms such as changes in tracer breakdown rate or changes in the tracer blood input function resulting from administration of CBD would not be sufficient to explain the changes in [^18^F]MeFWAY signals across various time points. Further, results from our behavioral studies are consistent with the anxiolytic action of CBD through 5-HT_1A_ receptor activation in the MIA-induced OA animal model.

The repeated low-dose CBD (5 mg/kg/day, s.c.)(De Gregorio et al., 2019) was selected based on a previously calculated value derived from the lowest i.v. acute dose (0.10 mg/kg) able to produce a significant decrease in 5-HT neuronal activity, taking into account the pharmacokinetic properties of CBD: Cmax, Tmax, and T1/2.(Samara et al., 1988; Mechoulam et al., 2002; Huestis, 2005; Deiana et al., 2012) This dosing regimen was similar to several other *in vivo* studies,(Parker et al., 2002; Parks et al., 2011; Rock et al., 2017) though higher doses ranging from 10–100 mg/kg have also been used.(Kwiatkowska et al., 2004; Tsang et al., 2008)

The CBD dose range on the neurochemical modulation may be target-dependent, may be different from those required for behavioral modulation, and may be sex-dependent.(Boyan et al., 2013) Further, the CBD dose effects may be varied for evoked vs. non-evoked pain. In our hands, there appeared to be a significant decrease in von Frey withdrawal threshold (e.g., increases in tactile pain responding) at the last post-surgical (and post-drug injection) time point(s) as measured by the electronic von Frey method, the manual monofilament test method had the same trend but was non-significant. Although a trend of increased anxiety or depressive-like behavior in the light/dark box or forced swim tests after OA induction, and decreased those behaviors after repeated low-dose CBD treatment was also observed, a bigger sample size or a higher dose than 5 mg/kg CBD (s.c.) is perhaps required to observe the antidepressant effect in the forced swim test.(Zanelati et al., 2010; Xu et al., 2019)

In general, based on our study results, the correlation of neurochemical changes (measured with the selective 5-HT_1A_ tracer [^18^F]MeFWAY) with the behavioral assessment was reasonable, with a better correlation of neurochemical changes with anxiety-like or depressive-like behavior, and less so with mechanosensory pain threshold measurement. These findings were consistent with literature data suggesting that repeated CBD treatment was able to prevent mechanical allodynia and anxiety-like behavior in rats experiencing neuropathic pain, but through different mechanisms. That is, TRPV1 channels would be required for the antiallodynic (but not the anxiolytic) effects of CBD, whereas 5-HT_1A_ receptors would be required for the anxiolytic (and to a lesser extent the antiallodynic) effects of CBD. Unfortunately, there is no radioligand available to date that directly targets TRPV1. Another confounding effect on mechanosensory pain threshold measurement in rodents with arthritis is that rodents are prey animals, and evolution has likely conditioned rodents to mask signs of disability and pain, making rodent gait compensations relatively more difficult to detect, even with assistance from videography.(Lakes and Allen, 2016) These compensatory patterns shared between left and right paws were observed in our studies. Further, in our hands, the delayed pain sensitivity increases post-surgery and into the post-drug injection period, particularly in the ipsilateral (right) paw, may have complicated the interpretation of the study results (i.e., the pain-threshold changes might result from either OA-induction or drug treatment, or both). We found the use of averaged (L and R) 50% paw withdrawal thresholds and final filament values were reasonably suitable for data comparison across various time points. Thus, Figures 9B and 9C are presented by using averaged (L and R) paw 50% withdraw threshold (g) and pooling all data from baseline and OA (under the same paradigm) and separate them based on the different drug treatment.

For the future pain assessment in OA model, Catwalk gait analysis, a robust test and relatively insensitive to extraneous stressors and insults, yet sensitively measures a form of functional/adaptive/dynamic weight bearing that is known to be impacted by OA, may be considered.(Jacobs et al., 2014; Carcole et al., 2019)

As to the potential confounding issue related to forced swim tests (FST), i.e., repeated FST assessment can result in behavioral carryover, typically a progressive increase in immobility across sessions, as we observed. This interpretation has been under debate because a progressive increase in floating (immobility) over time may reflect an adaptive learned behavioral response promoting survival, and not depression.(Molendijk and de Kloet, 2015) A recent study showed that 5 d repeated forced swim stress test (5d-RFSS) increased floating behavior over time but, importantly, did not induce emotional, homeostatic, or psychomotor symptoms, suggesting the test might have predictive validity to identify novel antidepressant treatments.(Mul et al., 2016) Further, we chose our within-subject experimental design to afford us power to examine whether CBD treatment could differentially affect this trajectory of immobility upon exposure to the forced swim test stressor, in conjunction with other behavioral and brain imaging measures.

Sex differences in 5-HT_1A_ receptor may reflect biological distinctions in the serotonin system contributing to sex differences in the prevalence of psychiatric disorders such as depression and anxiety. It has long been recognized that women are twice as likely to suffer from affective illnesses such as depression or anxiety than are men, while the reason for this sex difference is still unclear. Previous PET studies have investigated sex-based differences in 5-HT_1A_ binding, but the results are inconclusive; showing either no sex differences in 5-HT_1A_ receptor binding, or higher receptor binding for women. Various factors, such as different species (e.g., human (Jovanovic et al., 2008) or non-human primate(Wooten et al., 2013), different radiotracers with different affinities ([carbonyl-^11^C]WAY-100635) vs. [^18^F]MeFWAY), and different data analysis methods, may have led to the different outcomes, let alone an effect of sex-specific differences in endogenous serotonin levels that is not yet known. In our hands, female mice displayed significantly lower 5-HT_1A_ availability based on *in vivo* PET imaging (lower [^18^F]MeFWAY tracer binding, derived from avg. 17 brain ROIs), as compared to male mice (p <0.000), which is in line with the higher prevalence of affective disorders in women since lower levels of 5-HT_1A_ are associated with depression and/or anxiety.

Upon a closer look, it appeared that some regions more than others showed greater differences between CBD and saline-treated groups. This is conceivable since different brain regions with different density of 5-HT_1A_ may perceive different effects after CBD vs. saline treatment. In addition to global regional differences across different states, the potential sex-dependent regional differences would be of interest. In light of the limitation on PET imaging of the small brain structure in small animals, a future translational study in humans, having bigger brain structure and more reliable regional delineation and segmentation, will be needed to confirm these interesting findings.

The purpose for plotting out the male and female behavioral data both combined and separately is to emphasize the importance of investigating the sex differences, not just report the entire population of both males and females without clearly indicating their differences. Our results seem to suggest that females are more sensitive to CBD treatment, with increased 5-HT_1A_ binding (OA_d20 vs. OA_C, *p* < 0.001, Figure 8) and higher degree of restoration in anxiety or depressive-like behaviors after CBD treatment.(Figure 10 and 11)

Further, imaging studies with other radioligands to elucidate the disconnection between the 5-HT_1A_ imaging study and the von Frey test, and behavioral studies with further pharmacological interventions to provide direct evidence for the involvement of 5-HT_1A_ receptors in the behavioral effects of OA and CBD, are needed.

## Conclusions

Our study confirms the interaction of CBD with serotonin 5-HT_1A_ receptor is responsible, at least in part, for its analgesic and anxiolytic effects in the MIA-induced OA animal model. Further, these results are clinically relevant, as CBD is known to exhibit few side effects, and they support the initiation of clinical trials testing the efficacy of CBD-based compounds for treating OA pain and comorbid mood disorders.

## Data Availability

The datasets used and/or analyzed during the current study are available from the corresponding author on reasonable request.

## Abbreviations

CBD: Cannabidiol
OA: Osteoarthritis
5-HT: Serotonin
5-HT_1A_: Serotonin 5-HT_1A_ receptors
PET: Positron emission tomography
i.v.: Intravenous
MIA: Monosodium iodoacetate
L/D: light/dark box tests
FST: Forced swim tests
[^18^F]MeFWAY: [^18^F]trans-MeFWAY
ROI: Regions of interest
SUV: Standard uptake values
SUVR: Ratio of SUV(ROI) / SUV(reference)
CB: Cerebellum
